# Loss of Schwann cell plasticity in chronic inflammatory demyelinating polyneuropathy (CIDP)

**DOI:** 10.1186/s12974-016-0711-7

**Published:** 2016-09-27

**Authors:** Abhijeet R. Joshi, Laura Holtmann, Ilja Bobylev, Christian Schneider, Christian Ritter, Joachim Weis, Helmar C. Lehmann

**Affiliations:** 1 Department of Neurology, University Hospital of Cologne, Cologne, Germany; 2 Center for Molecular Medicine Cologne, Cologne, Germany; 3 Department of Otorhinolaryngology, University Hospital Essen, Essen, Germany; 4 Institute of Neuropathology, RWTH Aachen University, Aachen, Germany

**Keywords:** CIDP, GM-CSF, Chronic denervation, Schwann cell plasticity

## Abstract

**Background:**

Chronic inflammatory demyelinating polyneuropathy (CIDP) is often associated with chronic disability, which can be accounted to incomplete regeneration of injured axons. We hypothesized that Schwann cell support for regenerating axons may be altered in CIDP, which may account for the poor clinical recovery seen in many patients.

**Methods:**

We exposed human and rodent Schwann cells to sera from CIDP patients and controls. In a model of chronic nerve denervation, we transplanted these conditioned Schwann cells intraneurally and assessed their capacity to support axonal regeneration by electrophysiology and morphometry.

**Results:**

CIDP-conditioned Schwann cells were less growth supportive for regenerating axons as compared to Schwann cells exposed to control sera. The loss of Schwann cell support was associated with lower levels of granulocyte-macrophage colony-stimulating factor (GM-CSF) in CIDP sera and correlated with altered expression of c-Jun and p57kip2 in Schwann cells. The inactivation of these regulatory factors resulted in an altered expression of neurotrophins including BDNF, GDNF, and NGF in CIDP-conditioned Schwann cells in vitro.

**Conclusions:**

Our study provides evidence that pro-regenerative functions of Schwann cells are affected in CIDP. It thereby offers a possible explanation for the clinical observation that in many CIDP patients recovery is incomplete despite sufficient immunosuppressive treatment.

**Electronic supplementary material:**

The online version of this article (doi:10.1186/s12974-016-0711-7) contains supplementary material, which is available to authorized users.

## Background

Chronic inflammatory demyelinating polyneuropathy (CIDP) is an immune-mediated peripheral neuropathy that is pathologically characterized by endoneural inflammation, segmental demyelination, and axonal degeneration. Despite effective immunosuppressive treatment, CIDP patients often experience progressive impairment or improvement to a poorer functional level after a relapse [1–4]. Electrophysiological parameters indicate that the poor clinical recovery in those patients depends on the degree of axonal injury and their incomplete regeneration [5].

The regeneration of injured axons in the peripheral nervous system is essentially supported by Schwann cells, which trans-differentiate from myelinating to growth supportive Büngner repair cells. Thereby, Schwann cells change their morphology, down-regulate myelin genes and, at the same time, up-regulate genes that are responsible for promoting axon growth, neuronal survival, and macrophage invasion [6, 7]. Those include various neurotrophins like brain-derived neurotrophic factor (BDNF), glial cell line-derived nerve growth factor (GDNF), and nerve growth factor (NGF) [8]. This phenotypic switch of Schwann cells is controlled by a set of regulatory factors including positive regulators of myelination like Krox-20, Oct-6, Sox-10, and negative regulators like c-Jun, p57kip2, and Sox-2 [9–12].

We hypothesized that inflammatory mediators could diminish the pro-regenerative function of Schwann cells in CIDP, which may account for the axonal loss and hence incomplete clinical recovery in this condition. Assessing the function of Schwann cells in CIDP is an intricate task, because suitable transgenic mouse models are lacking and ethical and methodological issues prevent the direct use of patient-derived Schwann cells. We therefore adopted an animal model that allowed us to assess the plasticity of Schwann cells that were exposed to the complex milieu of inflammatory mediators specific for this condition.

## Methods

### Human sera

Sera samples were obtained from 14 CIDP patients (mean age 62 ± 13 years, ratio f:m = 3:11). All CIDP patients were diagnosed according to diagnostic criteria developed by the Peripheral Nerve Society (category probable or definite CIDP) [13]. Eight of 14 CIDP patients were on IVIg treatment, and sera were collected immediately before next application. Control sera (mean age 52 ± 19 years, ratio f:m = 6:9) were obtained from 15 individuals either healthy (*n* = 6) or patients (*n* = 9) with other neurological diseases including mild cognitive impairment/dementia (*n* = 3), Parkinson’s disease, multiple sclerosis, epilepsia, peripheral facial nerve paralysis, subarachnoid hemorrhage, and somatoform disorder (*n* = 1 each). All patients gave written informed consent. This study was approved by the local ethics committee of the medical faculty of the University of Cologne.

### Chronic denervation model and Schwann cell transplantation

**Fig. 1:**
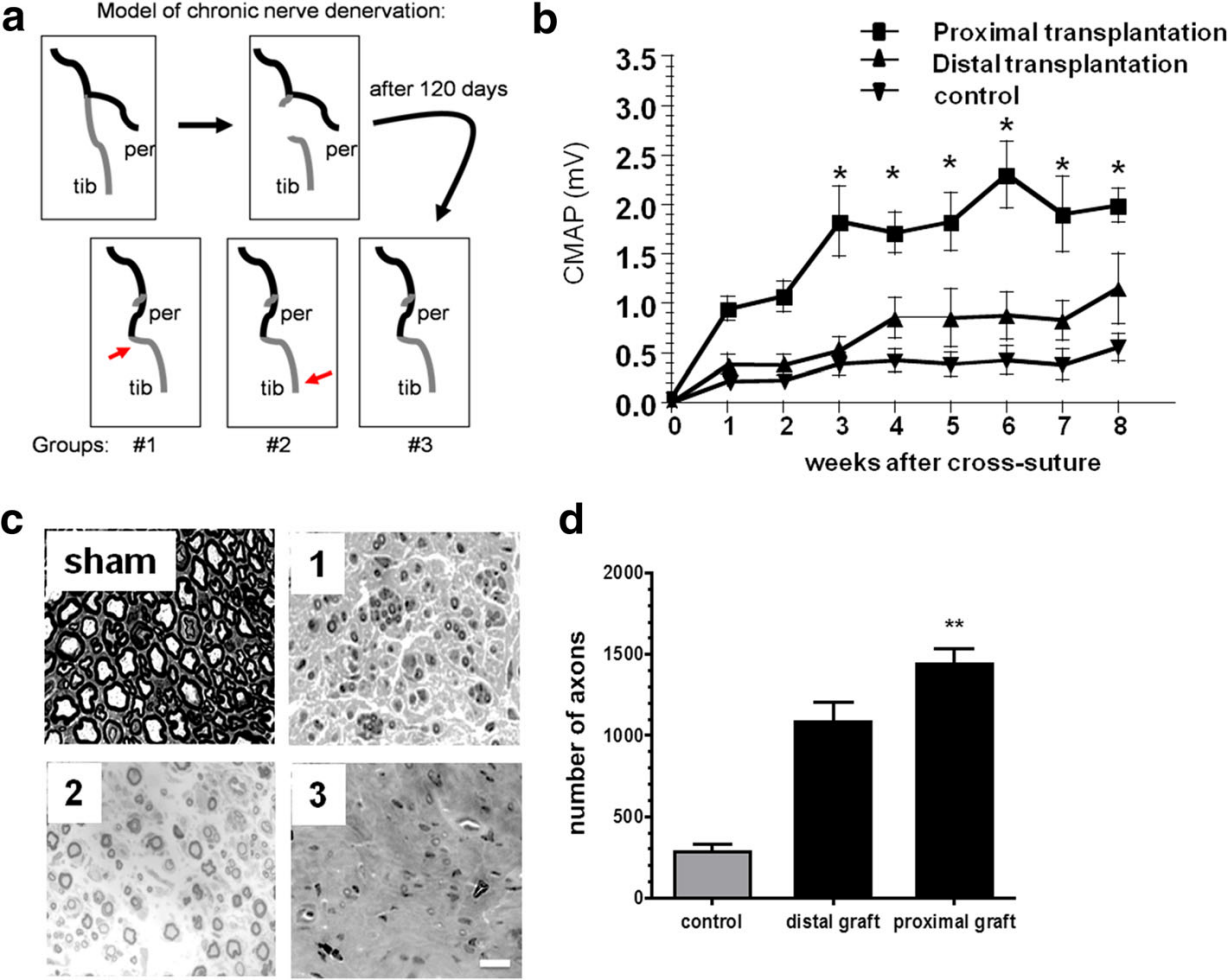
**a** Model of chronic denervation. The tibial nerve was transected and underwent chronic denervation. After 120 days, the common peroneal nerve was cut and the proximal segment of the peroneal nerve was ligated to chronically denervated tibial nerve. Schwann cells were transplanted at the sites shown by the *arrows* (*tib* tibial nerve, *per* common peroneal nerve). **b** Animals with Schwann cells transplanted at the proximal site show higher CMAP amplitudes compared to distal transplanted and control animals. **c** Semi-thin sections show myelinated axons in non-denervated (sham), denervated, and proximal Schwann cell transplant (*1*), denervated and distal Schwann cell transplant (*2*), and denervated and no Schwann cell transplant (*3*) nerves. **d** Graph summarizes the number of myelinated axons. *n* = 5–8 rats each group, **p < 0.01, *p < 0.05 (two-way ANOVA and Student’s *t* test) (*bar* = 20 μm)

All animal procedures were in accordance with the German Laws for Animal Protection and were approved by the local animal care committee and local governmental authorities. Ten-week-old female Wistar rats weighing 150–200 g were used. A graphical illustration of surgery procedures is depicted in Fig. 1a. Axons in the sciatic nerve separate distally into tibial nerve and common peroneal nerve. The tibial nerve was transected distal to the site of separation of the sciatic nerve into branches. The proximal stump of the cut nerve was ligated to the nearby gastrocnemius muscles in order to prevent its regeneration for 120 days (±2 days). Within the period of 120 days of chronic denervation, Schwann cells were cultured from neonatal rats. Lack of regeneration was confirmed by absence of compound muscle action potentials (CMAP) after stimulating the sciatic nerve. After 120 days, the second surgery was performed. The common peroneal nerve was transected, and the proximal stump of peroneal nerve was sutured to the distal stump of chronically degenerated tibial nerve. Immediately after suturing, approximately 1 million Schwann cells were transplanted into the nerve either in close proximity to the suture or distal to the suture (2 cm above the ankle). Animals in the control group were operated in the same way but received the same volume of cell medium instead of cell containing suspension. After establishment of the model, similar experiments were performed using Schwann cells treated either with control sera or CIDP sera with or without exogenous granulocyte-macrophage colony-stimulating factor (GM-CSF).

### GFP labeling of Schwann cells and transplantation

Schwann cells were transfected by means of the Lonza nucleofector II device (Lonza, Basel, Switzerland). Briefly, cells were harvested and 5,000,000 cells were pulsed using the basic glial cell nucleofection kit (Lonza) and 1 μg of pmaxGFP plasmids (Lonza). Cells were plated on PDL-coated petri dishes. Three days after transfection, cells were detached from dishes and counted. In a total of 10 μl Schwann cell medium, 1,000,000 cells were resuspended and injected into the freshly re-anastomosed nerve using a handheld Hamilton syringe. Medium without Schwann cells was injected as control. For detection, tibial nerve segments were cut 3 mm distal to the suture site and immersion-fixed overnight. Ten-micrometer transverse sections of nerve segments were cut and immunostained with antibodies against S100 (Sigma, 1:500) or GFP (Abcam, 1:350).

### Schwann cell culture and treatments

Schwann cells were prepared by a modified Brockes method [14]. Briefly, neonatal rats were anesthetized and sacrificed. Sciatic nerves were dissected out from the rats. Nerves were digested with 0.1 % collagenase for 30 min at 37 °C and then with 0.25 % trypsin for 1 h. Tissue was triturated and plated in DMEM/F12 medium containing 10 % FBS, 5 μg/ml bovine pituitary extract, 2.5 μM forskolin, and penicillin/streptomycin. To eliminate fibroblasts, the cells were treated with two cycles of cytosine arabinoside (10 μM) followed by complement lysis with anti-thymidine 1.1 antibody. In the resulting cultures, Schwann cells had a purity of more than 95 %. Human Schwann cells were obtained commercially (#1700, ScienCell Research Laboratory) and were cultured according to the instructions of the supplier. For in vitro experiments, Schwann cells were cultured in 24-well plates. CIDP and control sera were deactivated by heating at 55 °C for 30 min. Schwann cells were treated with deactivated sera (1:10) either with or without recombinant rat GM-CSF (10 ng/ml, #SRP3271, Sigma). Purified IgG fractions from same CIDP patients were extracted by ion-exchange chromatography as described [15]. Schwann cells were treated with pure IgG fractions (1:10).

### Fluorescence microscopy

Nine days after treatment with sera, Schwann cell cultures were fixed with 4 % PFA for 15 min, permeabilized with 0.2 % Triton X-100, and incubated with fluorescein-labeled phalloidin (P5282, Sigma) or primary antibody against S100 antibody (Sigma, 1:500) overnight at 4 °C. Cells were incubated with respective secondary antibody to tag S100. Cell nuclei were stained with Hoechst dye. Images of Schwann cells were acquired using a Keyence BZ9000 fluorescence microscope.

### qPCR

RNA was extracted from in vitro Schwann cells 3 days after treatment with sera using RNeasy plus mini kit (Qiagen), followed by reverse transcription of mRNA to cDNA using QuantiTect reverse transcription kit (Qiagen). Quantitative real-time polymerase chain reaction (qPCR) was carried out using a Rotor Gene 2000 (Corbett Life Sciences) system. Primers for specific genes (p57kip2_rat, c-jun_rat, GDNF_rat, BDNF_rat, NGF_rat, p57kip2_human, c-Jun_human, GAPDH_rat, GAPDH_human) were designed online with Primer3 (Whitehead Institute, Cambridge, USA) (Additional file 1: Table S1). GAPDH was used as a housekeeping gene. The relative gene expression was measured by comparative CT method.

### ELISA arrays

Serum concentrations of various cytokines were determined by commercially available ELISA kit (#EA-4002, Signosis). Concentration of GM-CSF in total 14 CIDP sera and 15 control sera were determined using GM-CSF ELISA array kit (#ELH-GM-CSF-001, RayBiotech) according to the manufacturer’s instructions. The minimum detectable amount of GM-CSF is less than 2 pg/ml.

### Electrophysiology

CMAP amplitudes were recorded by needle electrode insertion in the hindpaw (sole) and stimulation at the sciatic notch using a PowerLab signal acquisition set-up (AD Instruments) under controlled body temperature, as described [16]. CMAP recordings were done immediately before and in weekly intervals after nerve suturing.

### Morphometry

Tibial nerves were cut 15 mm distal to the suture site and immersion-fixed overnight. Nerve segments were embedded in epon, and 1-μm cross sections were stained with toluidine blue, as described [6]. All myelinated axons in a single whole cross section of the nerve were counted for quantification at light level (×40) and were analyzed using ImageJ software.

### Statistical analyses

Data were statistically analyzed using GraphPad Prism 5.0 (GraphPad Software). All numerical results are presented as means ± SEM. Differences between groups were compared with ANOVA and Student’s paired or unpaired *t* test as appropriate. *P* < 0.05 was considered statistically significant.

## Results

### Establishment of an in vivo model for Schwann cell transplantation after chronic denervation

**Fig. 2:**
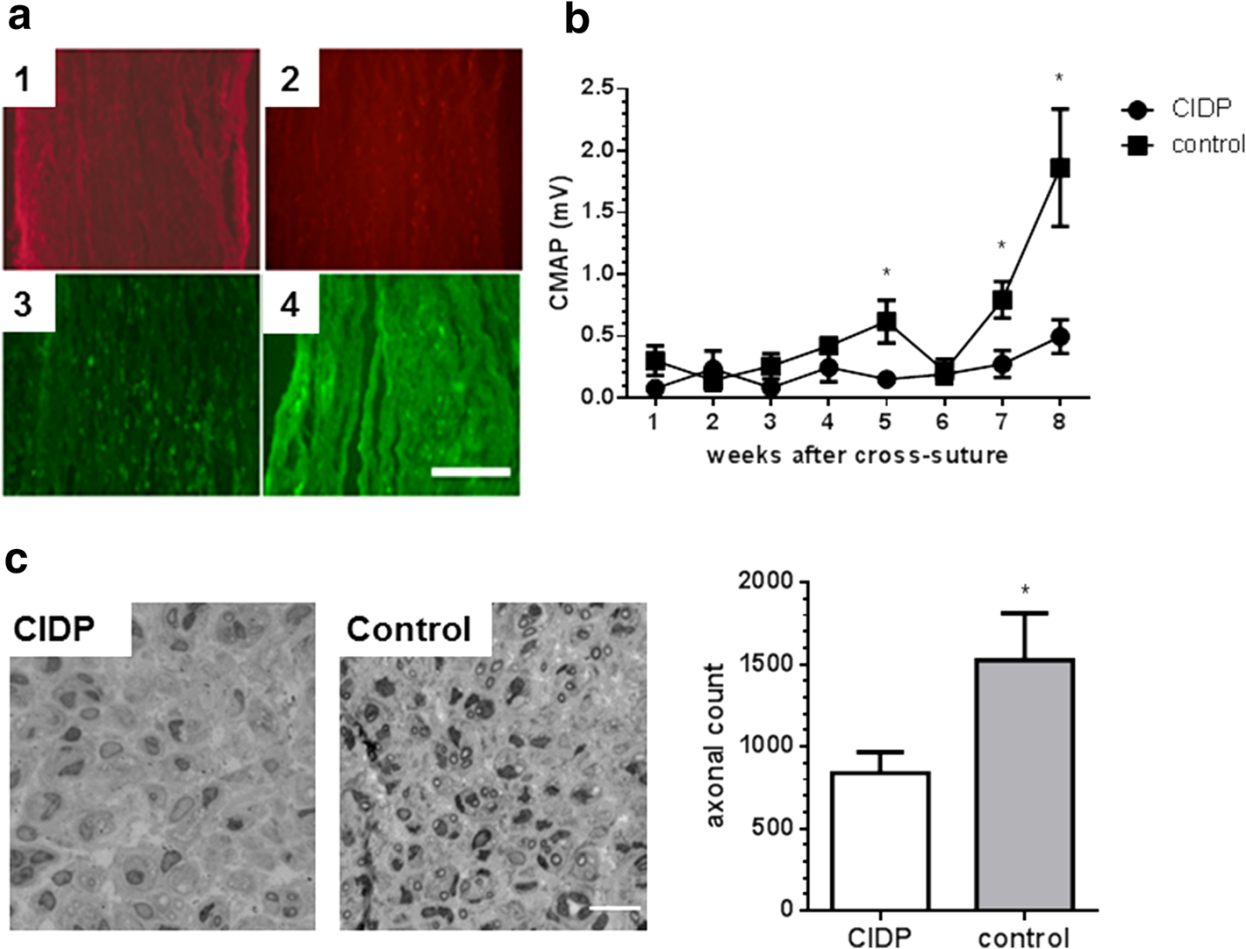
**a** Longitudinal sections of nerve sections two weeks after CFP-labeled Schwann cell grafting. 1: control nerve after staining with S100. 2: nerve section with Schwann cells graft after S100 staining. 3: nerve sections show spotty distribution of GFP-labeled Schwann cells. 4: staining of GFP-labeled Schwann cells with an antibody against GFP (*bar* = 20 μm). **b** Effect of transplantation of Schwann cells treated with CIDP or control sera on denervated nerve stumps. Transplantation of Schwann cells treated with CIDP sera (*circles*) resulted in slower and lower CMAP amplitude generation in rats compared to control (*squares*). **c** Semi-thin sections of nerves show significantly less number of regenerating axons in nerves from rats transplanted with CIDP treated Schwann cells compared to control. The graph shows quantification of all myelinated regenerated axons. *n* = 5 rats each group *p < 0.05 (ANOVA and independent *t* test) (*bar* = 20 μm)

To assess the pro-regenerative effects of Schwann cells in vivo, we adopted an animal model of axonal regeneration into a chronically denervated nerve stump [17]. In this model, the tibial nerve was first transected and the two segments remained separated for 120 days. During this time period, Schwann cells in the distal tibial nerve segment become atrophic and lose their ability to support axonal regeneration. Subsequently, a cross-suture of the freshly cut peroneal nerve to the tibial nerve stump was carried out. The transplantation of Schwann cells or cells with Schwann cell characteristics [18] into the distal nerve stumps can partially improve axonal regeneration into those nerve segments. For establishment of this in vivo model, we explored two different graft sites (immediately distal to the anastomosis versus far distal stump, *n* = 8 each group) because it is not known which transplantation site yields the highest benefit for promoting axonal regeneration in this model (Fig. 1a). Electrophysiological studies over 8 weeks after suturing both nerve ends showed significantly higher CMAP amplitudes recorded from rats with proximally transplanted compared to distally transplanted Schwann cells and control animals (Fig. 1b). Likewise, semi-thin sections 3 mm distal to the lesions site 8 weeks after re-suture showed significantly more regenerating axons in rats with proximally transplanted Schwann cells compared to controls (Fig. 1c, d). Two weeks after resuturing, only very few of the transplanted GFP-labeled Schwann cells were found by immunohistochemistry and anti-GFP immunostaining (Fig. 2a). Thus, we concluded that the majority of the transplanted Schwann cells did not survive for longer than 2 weeks, providing a possible explanation why proximal but not distal grafting enhances regeneration.

Altogether, the results demonstrated that the most growth-promoting effect of transplanted Schwann cells was observed when cells were transplanted proximally to the lesion site. Therefore, we used the same model for exploring the growth-promoting effects of Schwann cells exposed to human sera.

### Exposure to CIDP sera impairs the pro-regenerative function of Schwann cells

We hypothesized that inflammatory mediators in CIDP sera might alter the functional capacity of Schwann cells to assist nerve regeneration in vivo. Therefore, Schwann cells were treated with either CIDP or control sera and subsequently transplanted into chronic denervated nerve stumps. Electrophysiological studies showed that transplantation of CIDP exposed Schwann cells yielded significantly lower CMAP amplitudes compared to animals in which control sera exposed Schwann cells were grafted (Fig. 2b, *n* = 5 each group). The total number of regenerated axons in the CIDP group was significantly lower compared to the control group (Fig. 2c, *n* = 5 each group). Thus, we concluded that CIDP sera alter the function of Schwann cells in a way that they are less supportive for nerve regeneration in vivo.

### CIDP sera alter morphology and expression of transcription factors in Schwann cells

**Fig. 3:**
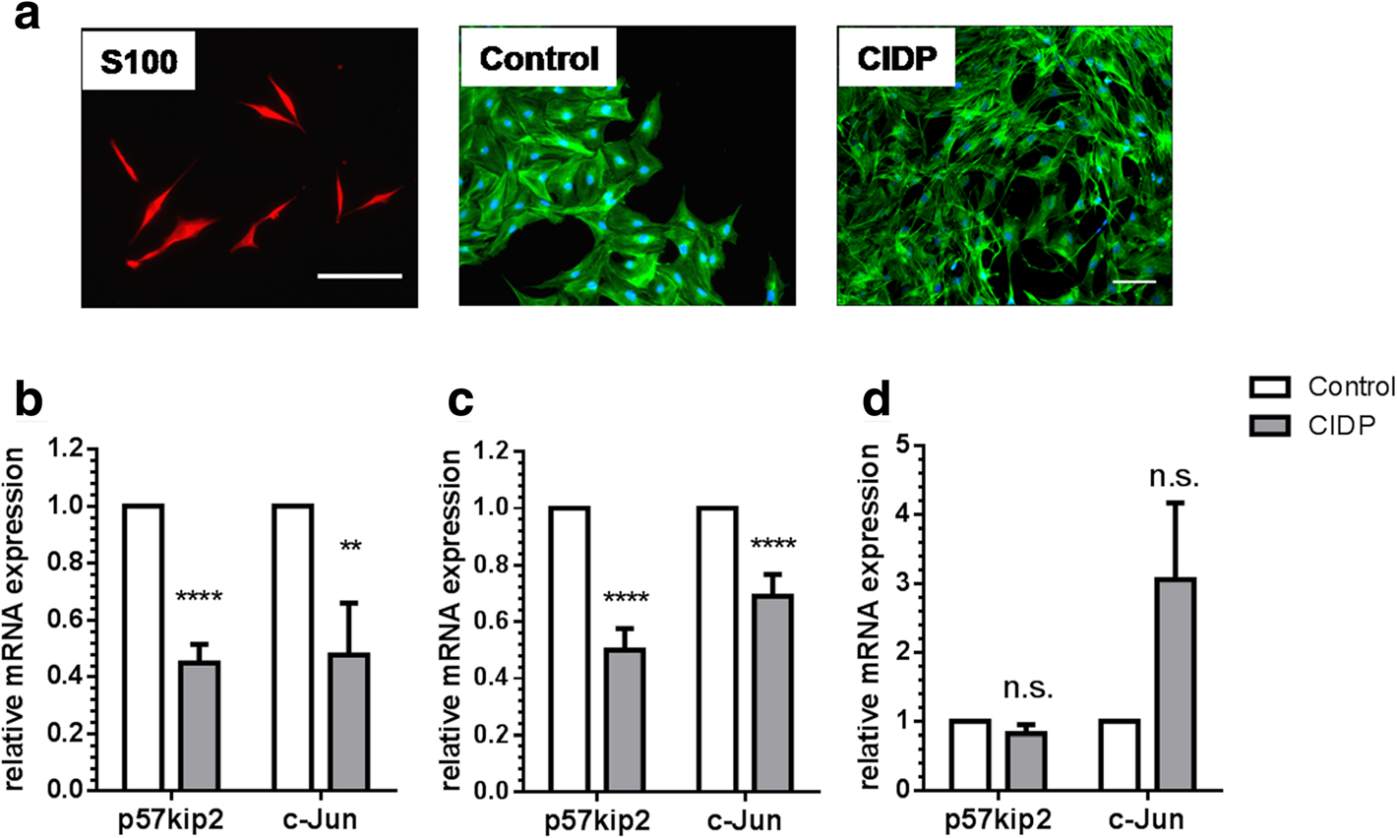
**a** Fluorescent staining of Schwann cells with S100 (S100) and phallodin (control and CIDP) stain. Staining with phalloidin shows the change in morphology of Schwann cells without (control) and with (CIDP) treatment with CIDP. Note morphological differences between phallodin and S100 staining. **b** Relative mRNA expression of p57kip2 and c-Jun in rat Schwann cells treated with CIDP sera. P57kip2 and c-Jun are significantly down-regulated after treatment with CIDP sera. **c** Relative mRNA expression of p57kip2 and c-Jun is decreased in human Schwann cells after treatment with CIDP sera. Human Schwann cells showed significant down-regulation to a similar extent after treatment with CIDP sera. **d** There is no significant down-regulation after treatment with IgG fractions from sera. GAPDH was used as a housekeeping gene. **p < 0.01, ****p < 0.0001 (unpaired Student’s *t* test) (*scale bar* = 40 μm)

To examine whether CIDP sera affect the morphology of Schwann cells in vitro, we treated the cell cultures with different CIDP sera. Schwann cells were first stained with an antibody against S100, thereby showing typical morphology of Schwann cells in vitro. Fluorescence staining of F-actin polymers with phalloidin, which stains all actin cytoskeleton [19–21], showed that Schwann cells treated with CIDP sera showed more elongated filopodial extensions and poor intercellular connections (Fig. 3a), resembling the non-growth supporting phenotype [10]. The de-differentiation of myelinating Schwann cells into a growth supportive phenotype is associated with dynamic regulation of p57kip2 and c-Jun gene expression [10, 11, 22, 23]. Schwann cells that were exposed to CIDP sera showed a significantly reduced mRNA expression of p57kip2 and c-Jun compared to Schwann cells exposed to control sera in vitro (Fig. 3b). To exclude the possibility that this effect is caused by the use of cells from another species (rodent), we also analyzed the expression of p57kip2 and c-Jun in a human Schwann cell line and found a similar down-regulation of these factors after exposure to CIDP sera (Fig. 3c).

### IgG fractions from CIDP sera do not affect mRNA expression of Schwann cell marker genes

Because autoantibodies are assumed to play a crucial role in the pathogenesis of CIDP, we then treated Schwann cells with purified IgG fractions from CIDP patients. Schwann cells treated with IgG from CIDP sera showed no change in the expression of p57kip2 and c-Jun (Fig. 3d). Therefore, we concluded that instead of IgG, other soluble factors in the sera are responsible for lower regenerative ability of Schwann cells.

### Low GM-CSF levels in CIDP sera are associated with reduced c-Jun and p57kip2 expression in Schwann cells

**Fig. 4:**
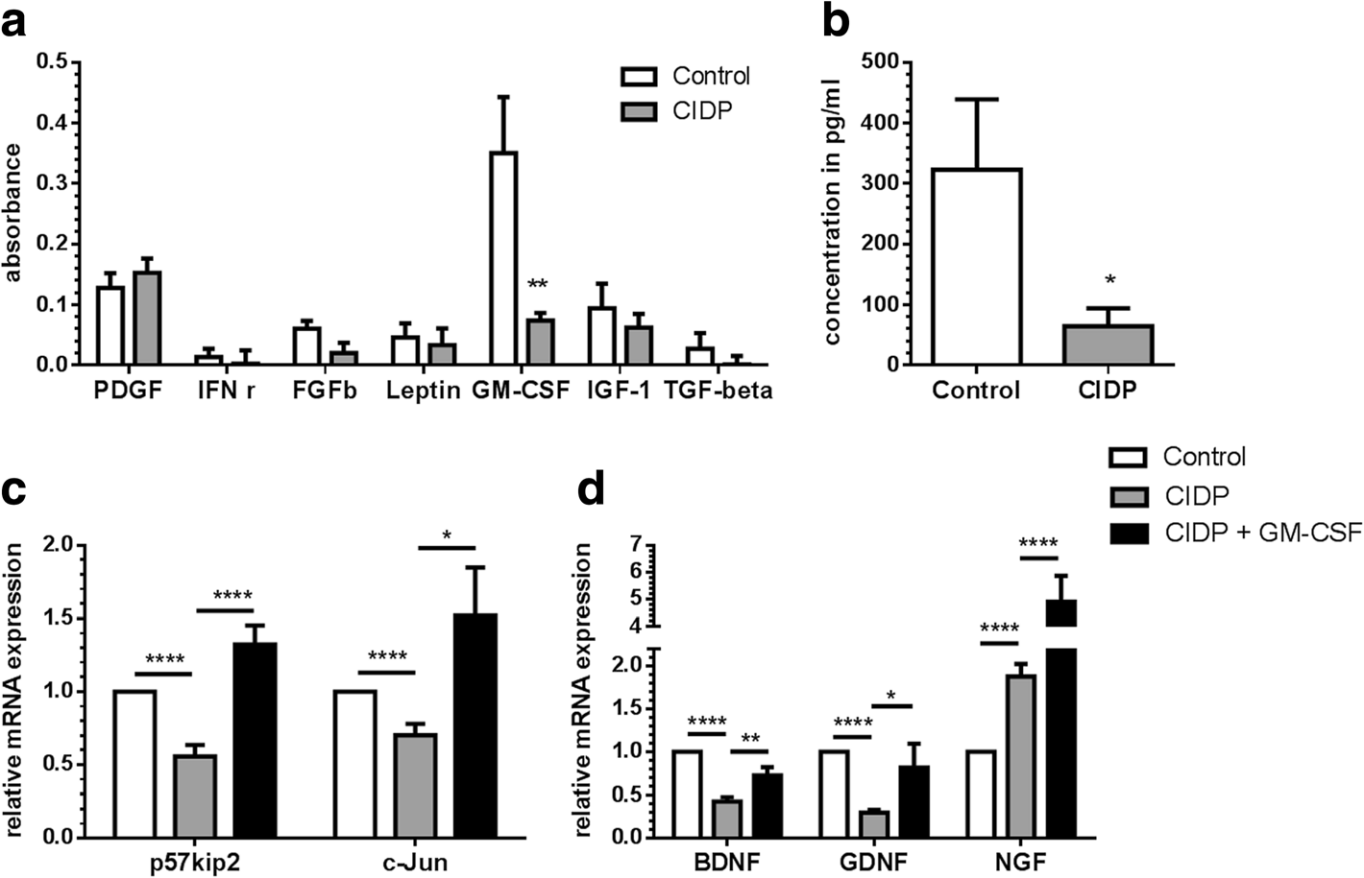
Measurement of cytokine expression in sera. Significantly lower expression of GM-CSF in CIDP sera compared to control sera was observed by cytokine ELISA array (**a**) which was confirmed by specific GM-CSF ELISA array (**b**). The effect of lower GM-CSF expression on gene expression was verified by adding recombinant rat GM-CSF to the Schwann cells treated with CIDP sera. **c** Addition of GM-CSF to the Schwann cells reversed the effect of CIDP sera on gene expression of p57kip2 and c-Jun. **d** CIDP-conditioned Schwann cells show significantly lower expression of BDNF and GDNF and higher expression of NGF. Down-regulation of BDNF and GDNF was reversed by addition of GM-CSF whereas expression of NGF was further increased by GM-CSF. GAPDH was used as a housekeeping gene for qPCR. *p < 0.05, **p < 0.01, ****p < 0.0001 (ANOVA and unpaired Student’s *t* test)

Since our results indicate that a soluble serum factor alters the expression of p57kip2 and c-Jun in Schwann cells, we further investigated the levels of a total of 31 soluble cytokines in CIDP sera. A complete list of cytokines along with their expression values is provided as a table in Additional file 1: Table S2. A cytokine array revealed significantly lower levels of GM-CSF in CIDP sera compared to control samples (Fig. 4a). These results were subsequently validated by a GM-CSF ELISA in 14 CIDP serum samples and 15 control samples (Fig. 4b). In order to confirm a causative relationship between GM-CSF and altered transcription levels, we treated Schwann cells with CIDP sera plus recombinant rat GM-CSF. As shown in Fig. 4c, expression of p57kip2 and c-Jun were induced and were reversed to normal levels after the addition of GM-CSF.

### CIDP sera treated Schwann cells show altered expression of growth factors

In order to investigate whether lack of regenerating support from Schwann cells is the result of altered expression profile of various growth factors, we assessed gene expression of BDNF, GDNF, and NGF. Expression of BDNF and GDNF was decreased significantly in CIDP treated Schwann cells and was increased after addition of exogenous GM-CSF (Fig 4d). In contrast, expression of NGF was increased by addition of CIDP sera which was further increased by exogenous GM-CSF (Fig. 4d).

### Exogenous GM-CSF does not reverse diminished pro-regenerative ability of Schwann cells in vivo

**Fig. 5:**
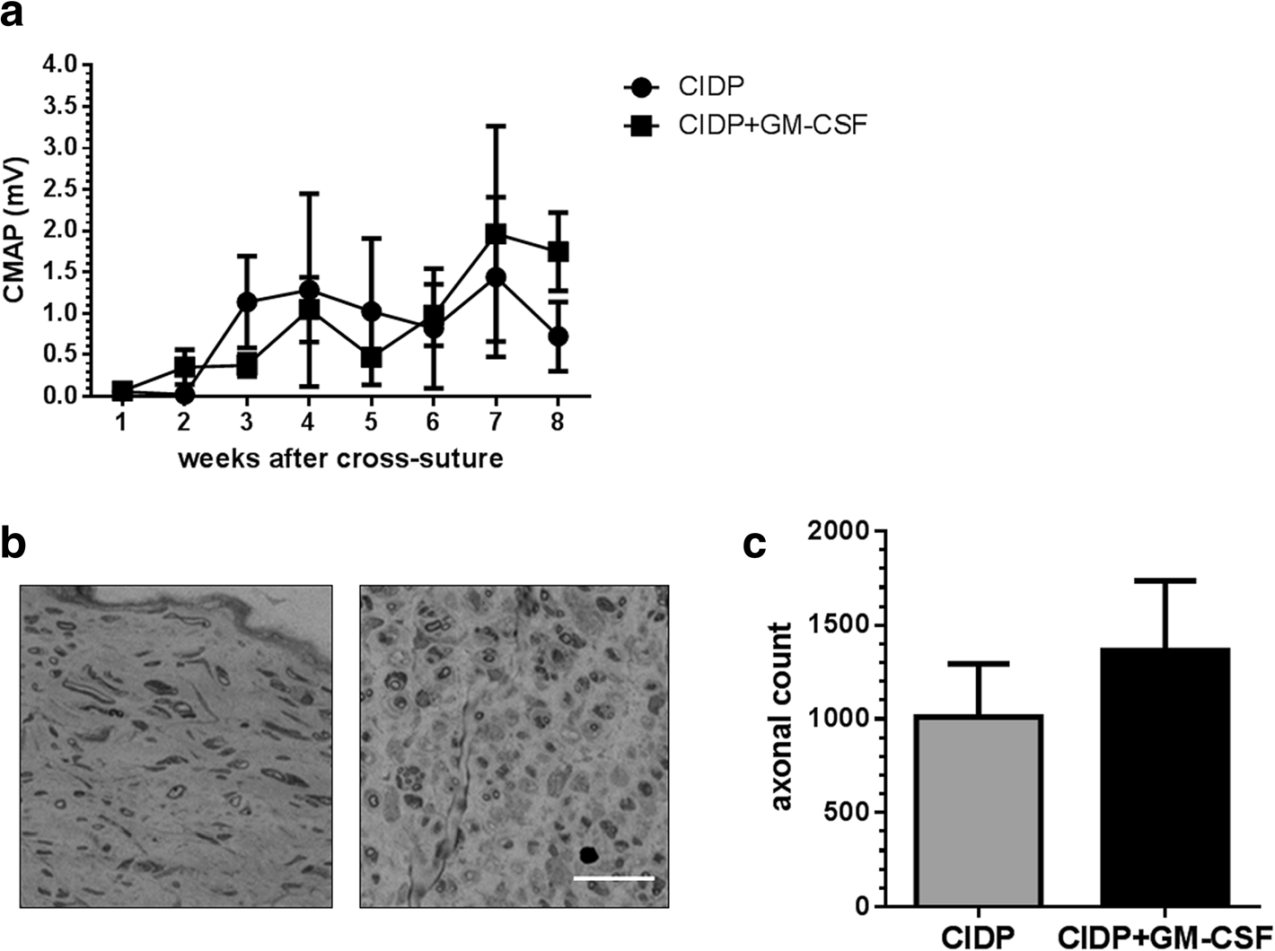
Effect of exogenous GM-CSF on the ability of CIDP-conditioned Schwann cells to support axonal regeneration. The first group of rats received Schwann cells conditioned with CIDP sera, and the second group received CIDP-conditioned and GM-CSF-treated Schwann cells. **a** Electrophysiological analysis showed no significant difference between two groups. **b** Morphometrical analysis was used to analyze number of regenerated axons in nerves. **c** The second group showed a modest increase in axon count which was not statistically significant. *n* = 3 rats each group (ANOVA and Student’s *t* test) (*bar* = 20 μm)

As our data indicated low levels of GM-CSF in CIDP sera, we hypothesized that addition of exogenous GM-CSF along with CIDP sera to Schwann cells might regain their pro-regenerative ability. Therefore, Schwann cells treated with CIDP sera and GM-CSF were transplanted in chronically denervated nerve. Electrophysiology measurements showed slight increased nerve regeneration in animals, which received GM-CSF conditioned Schwann cells; however, it did not reach statistical significance (Fig. 5a). In line with electrophysiology, morphometrical analysis showed slightly increased number of regenerating axons but not statistically significant (Fig. 5b, c).

## Discussion

Our study provides evidence that in CIDP, Schwann cells become dysfunctional to support regeneration of injured axons. It offers therefore a possible explanation for the clinical observation that many patients with CIDP do not improve, despite adequate immunosuppressive treatment. Electrophysiological studies in humans are consistent with this concept, since they could demonstrate that the weakness in CIDP patients mostly correlates with electrophysiological parameters of axon loss rather than slowing nerve conduction velocities [24, 25].

As soluble factors that cause this partial Schwann cell dysfunction we found significantly reduced serum levels of the hematopoietic cytokine GM-CSF in CIDP sera. GM-CSF is expressed by a variety of inflammatory cells including T cells, monocytes, and macrophages [26]. The exact role of GM-CSF in the pathogenesis of CIDP is unknown. A previous study that included samples from eight CIDP patients did not find any difference in GM-CSF in the CIDP group compared to control samples [27]. Apart from a higher sample size, methodological differences including lack of a confirmatory ELISA in the study of Sainaghi and colleagues could provide an explanation for these discrepant findings. The expression of GM-CSF in nerve tissue in CIDP has not been studied and may not necessarily correlate with serum levels. However, there is evidence that GM-CSF belongs to a group of cytokines that plays an important role during Wallerian degeneration [28–30]. After nerve injury, fibroblasts express higher levels of GM-CSF to enhance myelin clearance by activation of macrophages and Schwann cells. As an extension to these reports, we found that GM-CSF modulates the expression of the two Schwann cell genes c-Jun and p57kip2. The results are thereby in line with findings that GM-CSF also regulates the expression of c-Jun in various tumor cell lines and leucocytes [31, 32]. C-Jun and p57kip2 are negative regulators of Schwann cell myelination [10, 11, 33], thereby preventing premature or hypermyelination. Recent studies demonstrated that c-Jun is also rapidly up-regulated after nerve injury and induces a molecular reprogramming of Schwann cells that results in de-differentiation from myelinating Schwann cells to a denervated, growth supportive phenotype [7, 9, 23, 33]. The importance of c-Jun for Schwann cell plasticity is further emphasized by findings that axonal regeneration after nerve injury is severely reduced in Schwann-cell-specific c-Jun knockout mice. Immunohistochemical studies from nerve and skin biopsies could also demonstrate that c-Jun is up-regulated in Schwann cells in a number of different neuropathic conditions including CIDP and Guillain-Barré syndrome [34]. Notably, the c-Jun expression profile in CIDP samples was quite heterogeneous, which points to a different and potentially dysregulated activity of c-Jun in some patients and/or Schwann cell populations.

In an attempt to further characterize the molecular phenotype of CIDP-conditioned Schwann cells, we analyzed the mRNA expression of different neurotrophins. Our in vitro data indicate that CIDP-conditioned Schwann cells express low levels of neurotrophins BDNF and GDNF. Both BDNF and GDNF are known to facilitate regeneration of motor neurons in chronically denervated nerves [35]. Moreover, deprivation of BDNF by Schwann cells impairs axonal regeneration and the myelination of regenerated fibers after nerve injury [36]. On the other hand, role of NGF in axonal regeneration of peripheral nerves is diverse. Whereas NGF is needed for regeneration of nociceptive and sympathetic axons [8], it has very little or no effect of regeneration of motor neurons [37]. In contrast, exogenous application of NGF delays the onset of regeneration after nerve injury [38]. This might explain why we did not observe pro-regenerative effect of higher NGF expression in CIDP-conditioned Schwann cells. How the expression of neurotrophins is regulated in Schwann cells is out of scope of this study. However, previous studies strongly demonstrate that expression of neurotrophins like BDNF and GDNF are regulated by c-Jun in Schwann cells [39]. Previous reports demonstrated that although chronically denervated Schwann cells are less supportive for axonal regeneration, they still maintain the ability to remyelinate the axons after regeneration [40]. Thus, transplanted Schwann cells might provide primary signal for initiation of regeneration, whereas endogenous Schwann cells might be the primary cells responsible for myelination once axonal regeneration is complete. Taken together, our study demonstrates that sera from CIDP patients diminish pro-regenerative ability of Schwann cells after axonal degeneration. Our in vitro data indicate that lack of GM-CSF cytokine in sera is one plausible cause for this effect.

A clear limitation of our animal model is that the complex experimental procedures increased the variability in outcome measures to an extent that prevented us to collect convincing in vivo evidence for this hypothesis. To investigate whether exogenous GM-CSF can reverse the diminished pro-regenerative ability of Schwann cells in vivo, we transplanted CIDP and GM-CSF conditioned Schwann cells in a small number of animals (*n* = 3 rats each group). Exogenous GM-CSF increased the axonal regeneration to some extent as assessed by electrophysiology and morphometry. However, this difference did not reach statistical significance. A power analysis derived from these data revealed that an unreasonably high sample size would be required to demonstrate statistical significant differences. Moreover, we cannot exclude the possibility of involvement of other cytokines and inflammatory mediators apart from GM-CSF for diminished Schwann cells’ growth supporting ability.

## Conclusions

In summary, our study provides evidence that the inflammatory environment in CIDP affects the support of non-myelinating Schwann cells for regenerating axons. This loss of Schwann cell plasticity is mediated by changes in transcription factors that normally induce a growth supportive Schwann cell phenotype after nerve injury. Our in vitro data indicate that GM-CSF may play a critical role for the comprised Schwann cell function in CIDP, but further work is required to determine the role of cytokines and other soluble serum factors in mediating loss of Schwann cell growth support in CIDP.

## Additional file

Additional file 1: Supplementary information. **Table S1**: Primer sequences. **Table S2**: List of cytokines and their expression. (DOCX 14 kb)

## Abbreviations

BDNF: Brain-derived nerve growth factor
CIDP: Chronic inflammatory demyelinating polyneuropathy
CMAP: Compound muscle action potential
ELISA: Enzyme-linked immunosorbent assay
GDNF: Glial cell line-derived neurotrophic factor
GM-CSF: Granulocyte-macrophage colony-stimulating factor
NGF: Nerve growth factor
qPCR: Quantitative real-time polymerase chain reaction
