# Supplementary material for: Loss of Schwann cell plasticity in chronic inflammatory demyelinating polyneuropathy (CIDP)
Source: J Neuroinflammation. 2016 Sep 27;13:255. doi: 10.1186/s12974-016-0711-7 (PMC5039906; doi:10.1186/s12974-016-0711-7)
Supplement: Additional file 1: — Supplementary information. Table S1: Primer sequences. Table S2: List of cytokines and their expression. (DOCX 14 kb) [file 12974_2016_711_MOESM1_ESM.docx]

**Supplementary information:**

Table S1: Primer sequences

| Gene | Sequence (5’-3’) |
| --- | --- |
| p57kip2_rat-forward | AGAGAACTTGCTGGGCATCT |
| p57kip2_rat-reverse | TAGGGACCAGCGTACTCCTT |
| c-Jun_rat- forward | CTCTCTCTCTCCCCAGCAAC |
| c-Jun_rat- reverse | GATCACAGCGCATGCTACTT |
| GDNF_rat- forward | CTGCCCGCCGGTAAGAGG |
| GDNF_rat- reverse | CGTCATCAAACTGGTCAGGATAATC |
| BDNF_rat- forward | CAAAAGGCCAACTGAAGC |
| BDNF_rat- reverse | CGCCAGCCAATTCTCTTT |
| NGF_rat-forward | CCCCGAATCCTGTAGAGA |
| NGF_rat-reverse | CACGCAGGCTGTATCTAT |
| GAPDH_rat-forward | CCTGTTCATCCCTCCACACATC |
| GAPDH_rat-reverse | CCAGTGATTTTCCAGCCCTAATC |
| p57kip2_human-forward | GCGGCGATCAAGAAGCTGTC |
| p57kip2_human-reverse | CCGGTTGCTGCTACATGAAC |
| c-Jun_human- forward | TCTCACAAACCTCCCTCCTG |
| c-Jun_human- reverse | GAGGGGGTTACAAACTGCAA |
| GAPDH_human-forward | ACCACAGTCCATGCCATCAC |
| GAPDH_human-reverse | TCCACCACCCTGTTGCTGTA |

Table S2: List of cytokines and their expression: All values are expressed as blank corrected absorbance values.

|  | CIDP sera | | Control sera | |
| --- | --- | --- | --- | --- |
| Cytokines | mean | SD | mean | SD |
| TNF alpha | undetected | | | |
| VEGF | undetected | | | |
| PDGF | 0.1529 | 0.0401 | 0.1281 | 0.0418 |
| IL-10 | undetected | | | |
| IFN r | 0.0022 | 0.0383 | 0.0137 | 0.0234 |
| EGF | undetected | | | |
| PLGF | 0.0144 | 0.0306 | 0.0319 | 0.0248 |
| FGFb | 0.0204 | 0.0287 | 0.0606 | 0.0224 |
| GCSF | undetected | | | |
| IL-6 | undetected | | | |
| b-NGF | undetected | | | |
| Leptin | undetected | | | |
| GMCSF | 0.0739 | 0.0218 | 0.3506 | 0.1613 |
| Resistin | 0.8463 | 0.1880 | 1.3999 | 0.3796 |
| SCF | undetected | | | |
| IGF-1 | 0.0624 | 0.0386 | 0.0945 | 0.0705 |
| IL-1a | 0.0672 | 0.0404 | 0.1425 | 0.0098 |
| PAI-1 | undetected | | | |
| MCP-1 | undetected | | | |
| TGF-beta | 0.0011 | 0.0244 | 0.0274 | 0.0442 |
| IL-8 | undetected | | | |
| IL-12 | undetected | | | |
| MIP-1a | undetected | | | |
| Adipokine | 2.5386 | 0.1386 | 2.4021 | 0.3929 |
| IP-10 | undetected | | | |
| IL-13 | undetected | | | |
| IL-2 | 0.3990 | 0.0335 | 0.6437 | 0.1101 |
| IL-17a | undetected | | | |
| Rantes | undetected | | | |
| Eotaxin-3 | undetected | | | |
| IL-4 | undetected | | | |
